# Pervasive brain monitoring and data sharing based on multi-tier distributed computing and linked data technology

**DOI:** 10.3389/fnhum.2014.00370

**Published:** 2014-06-03

**Authors:** John K. Zao, Tchin-Tze Gan, Chun-Kai You, Cheng-En Chung, Yu-Te Wang, Sergio José Rodríguez Méndez, Tim Mullen, Chieh Yu, Christian Kothe, Ching-Teng Hsiao, San-Liang Chu, Ce-Kuen Shieh, Tzyy-Ping Jung

**Affiliations:** ^1^Pervasive Embedded Technology Lab, Computer Science Department, National Chiao Tung UniversityHsinchu, Taiwan, R.O.C.; ^2^Swartz Center for Computational Neuroscience, University of CaliforniaSan Diego, CA, USA; ^3^Research Center for Information Technology Innovation, Academia SinicaTaipei, Taiwan, R.O.C.; ^4^National Center for High-performance ComputingHsinchu, Taiwan, R.O.C.

**Keywords:** brain computer interfaces, bio-sensors, machine-to-machine communication, semantic sensor web, linked data, Fog Computing, Cloud Computing

## Abstract

EEG-based Brain-computer interfaces (BCI) are facing basic challenges in real-world applications. The technical difficulties in developing truly wearable BCI systems that are capable of making reliable real-time prediction of users' cognitive states in dynamic real-life situations may seem almost insurmountable at times. Fortunately, recent advances in miniature sensors, wireless communication and distributed computing technologies offered promising ways to bridge these chasms. In this paper, we report an attempt to develop a pervasive on-line EEG-BCI system using state-of-art technologies including multi-tier Fog and Cloud Computing, semantic Linked Data search, and adaptive prediction/classification models. To verify our approach, we implement a pilot system by employing wireless dry-electrode EEG headsets and MEMS motion sensors as the front-end devices, Android mobile phones as the personal user interfaces, compact personal computers as the near-end Fog Servers and the computer clusters hosted by the Taiwan National Center for High-performance Computing (NCHC) as the far-end Cloud Servers. We succeeded in conducting synchronous multi-modal global data streaming in March and then running a multi-player on-line EEG-BCI game in September, 2013. We are currently working with the ARL Translational Neuroscience Branch to use our system in real-life personal stress monitoring and the UCSD Movement Disorder Center to conduct in-home Parkinson's disease patient monitoring experiments. We shall proceed to develop the necessary BCI ontology and introduce automatic semantic annotation and progressive model refinement capability to our system.

## Introduction

In recent years, electroencephalography (EEG) based brain computer interfaces (BCI) have left their laboratory cradles and began to seek real-world applications (Lance et al., 2012). Wearable BCI headsets such as Emotiv *EPOC*, NeuroSky *MindSet* and *MINDO* are selling as consumer products while applications such as silent communication using *The Audeo* by Ambient and focus/relax exercises using the *Mindball* by Interactive Productline are attracting widespread attention. Despite this hype, BCI applications still need to overcome a few basic challenges in order to become truly useful in real-world settings:

1. *Finding reliable ways to determine users' brain states:* it is well known that individuals' EEG responses exhibit significant differences even when the individuals perform the same task or exposed to identical stimuli. For example, the EEG correlates of fatigue vary remarkably across different subjects even though they remain relatively stable among different sessions of the same subject (Jung et al., 1997). As a result, long training sessions at different fatigue levels must be conducted on each user in order to calibrate a personalized EEG-based fatigue monitoring model. Hence, there is a pressing need to identify common EEG correlates of certain brain states in order to reduce the amount of training data required to calibrate individual users' BCI systems.
2. *Adapting prediction and classification models to track users' brain dynamics*: EEG responses are highly non-stationary due to rapid changes of users' brain conditions. Consequently, a model calibrated according to a user's initial condition may lose its accuracy over a prolonged session and must be adjusted periodically during that session based on real time analysis of the EEG and environmental data collected continuously by the BCI system. How to implement such a *progressive refinement* of brain state prediction and classification models remains an open question.
3. *Optimizing effectiveness of brain stimulation:* BCI systems often employ auditory, photic/visual, haptic, and vibrating stimuli to evoke users' EEG responses or modulate their brain states. Again due to users' brain dynamics and their habituation toward repetitive stimulation, the effectiveness of these stimuli often deteriorate and also affected by the changes in environmental conditions. Thus, feedback mechanisms must be in place to regulate the stimuli in order to counter the habituation trend and the environmental influences.

To tackle these challenges, real-world EEG-BCI systems not only need to conduct real-time signal analyses and brain state predictions on individual data set but also to perform data-mining and machine-learning operations over large data sets collected from vast user population over extended time periods. To do so, future EEG-BCI systems must be connected to high-performance computing servers as well as massive on-line data repositories through the global Internet in order to excavate the wealth of information buried in the massive data collection and adapt their prediction models and operation strategies in response to the incoming data in real time. To realize these futuristic scenarios, we implemented a pilot on-line EEG-BCI system using wireless dry-electrode EEG headsets and MEMS motion sensors as the front-end devices, Android mobile phones as the personal user interfaces, compact personal computers as the near-end Fog Servers and the computer clusters hosted by the Taiwan National Center for High-performance Computing (NCHC) to provide the far-end Cloud Computing services. So far, we have conducted two sets of experiments using our pilot system: first, a trial of synchronous multi-modal global data streaming was carried out in late March and then three runs of the multi-player on-line EEG-BCI game *EEG Tractor Beam* were played since late September, 2013. Outcomes of these experiments were discussed in the Results section.

This paper adopts the structure of a technology report. The Methods section expounds the two architectural concepts as well as the three operating scenarios of this system. The following Results section described the two pilot experiments performed during the past year and used them as the examples to explain the relatively easy and modular approach to use this system to develop novel applications. Finally, the Discussions section highlights the advantage of employing this system to implement future real-world EEG-BCI applications. It also discusses the information security and user privacy issues that may arise from the real-world deployment of this system. Potential cost/benefit tradeoffs are also considered. Since this is an on-going work to develop a pilot system, a list of future work is provided at the conclusion.

## Methods

This pervasive on-line EEG-BCI system was built upon two information and communication technologies: (1) a *multi-tier distributed computing infrastructure* that is based on Fog and Cloud Computing paradigms and (2) a *semantic Linked Data superstructure* that connects all the data entries maintaining in this distributed computing infrastructure through meta-data annotation. The system was designed to support three operation scenarios: (1) *“Big Data” BCI*, which can maintain ever-increasing amount of real-world BCI data in a scalable distributed data repository and search for data relevant to specific task and event types using semantic queries; (2) *Interactive BCI*, which enables the BCI systems to regulate their brain stimuli based upon real-time brain state prediction and feedback control; (3) *Adaptive BCI*, which can train and refine brain state prediction and classification models based on the relevant data sets gathered through semantic data queries and then push these models back to the EEG signal processing and brain state prediction pipelines in real time. Following sections offer a conceptual overview of the relevant technologies and the system operation. Engineering details, however, will be described in a complementary paper.

### Multi-tier Fog and cloud computing infrastructure

#### Rationale

Real-world BCI systems (as well as other personal telemonitoring systems) constantly face the daunting challenge of providing reliable long-term monitoring results in the ever-changing real-world situations using only battery-powered devices. As Cummings pointed out in her paper (Cummings, 2010), the necessary technology for hardware miniaturization and algorithmic improvement may not become available in the near future. Meanwhile, it is simply impossible to perform the computation and communication demanding tasks on these wearable systems: *computation offloading* provides the only viable solution, and the adoption of *Fog Computing* paradigm was the practical engineering approach we chose to tackle this challenge.

Fog Computing was first proposed by Bonomi of Cisco (Bonomi et al., 2012) as an *ad-hoc* distributed computing paradigm that utilizes computing resources available among on-line computers (known as the Fog Servers) close to the wireless sensors and the mobile phones to offload their computing burden so as to prolong their battery life and enhance their data processing performance. When we superimpose Fog Computing onto Cloud Computing, we created a three-tier distributed computing architecture with the Fog Servers serving as the near-end computing proxies between the front-end devices and the far-end servers. These near-end servers can offer potent data processing and storage services to the front-end devices while incurring minimal amount of communication latency. Thus, the Fog Servers can be useful aids in real-time human–computer interactions.

For the sake of reaping the most benefit from this three-tier architecture, however, one must allocate computing tasks strategically at each tier and exchange information efficiently between the tiers using succinct data formats and interoperable communication protocols. In the rest of this section, we explore various ways to trade off the computation and communication workloads among the front-end, near-end, and far-end computing nodes. Our objective is to optimize the computation and communication efficiency of the entire infrastructure while enhancing the responsiveness and robustness of the pervasive on-line EEG-BCI systems.

#### Architecture

**Figure 1:**
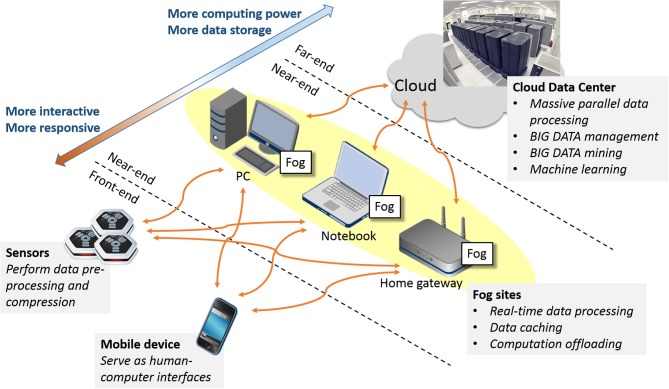
**Conceptual architecture of Fog/Cloud Computing infrastructure**.

Figure 1 illustrates the concept of multi-tier Fog and Cloud Computing. The first tier, known as the *front-end*, consists of battery-powered wireless sensors and mobile devices, which serve as the interfaces between the physical world, the human users and the cybernetic information infrastructure. The second tier or the *near-end* is formed by an *ad-hoc* conglomerate of consumer IT products such as personal computers, television set-top boxes, and game consoles close to the front-end devices over the Internet. These computing nodes, known as the Fog Servers, have sufficient electric power, data storage, and computing capacity to offload the computing burden from the front-end devices in order to prolong their battery lives and enhance their performance. The final tier or the *far-end* is made up of Cloud Servers installed in public or private data centers. These high-performance computers not only have plenty computing power, storage capacity and communication bandwidth; they have also accumulated vast amount of information and can use them to make deduction and prediction beyond the capability of stand-alone computers. This massive Cloud-based information warehouse and computing engine is the “backbone” of this distributed infrastructure. Sophisticated as it seems, the Fog/Cloud Computing infrastructure is expected to be widely deployed riding the tie of the Internet-of-Things. For examples, the smart homes and buildings will have smart electric meters that can control the power consumption of electric appliances while interacting with the smart power grids; the in-home multimedia servers will deliver bundled information and communication services from the “Internet cloud” to individuals' personal devices; intelligent transportation systems will install roadside controllers/servers that will interact with pedestrians' mobile phones and vehicles' on-board computers while pulling and pushing data to the municipal and national data centers. From this perspective, our on-line EEG-BCI systems can be regarded as a kind of pervasive personal telemonitoring system. Consequently, all our design decisions were made to ensure interoperability with the de-facto or emerging standards in the field of *machine-to-machine communication* and *Internet-of-Things*.

#### Computation and communication tradeoffs

Currently, there exist a communication bottleneck and an information chasm between the mobile applications running on the front-end devices and the computing services provided by the far-end Cloud Servers. The existence of the communication bottleneck is due to the fact that 3G/Wi-Fi Internet connections offer asymmetric data communication. These wireless networks operate based on the assumption that data flow in larger quantity and higher rates from the Internet content/service providers to the individual consumers; hence, the provider-to-consumer down-links are allotted much wider bandwidth than the consumer-to-provider up-links. However, the balance is gradually tilted by the increasingly widespread deployment of Internet sensors; in the near future, much more data will be generated by the front-end devices than the results produced by the far-end servers. Meanwhile, an information chasm is also created by the separation between the data producers (sensors) and the data processors (servers). The data transport latency through the Internet core can run between 200 and 500 ms. Thus, it is impossible for mobile applications to produce sub-second real-time responses using Cloud Computing. Along with other Fog Computing advocates, we therefore propose to disperse computing tasks along the data transport paths. Specifically, we suggest: (1) to install powerful embedded processors in wireless sensors in order to perform on-board data pre-processing and streaming analysis; (2) to convert personal computers, television set-top boxes, and game consoles into ubiquitous Fog Servers through the deployment of *ad-hoc* computing proxy software in order to perform most of the real-time computation; (3) to support meshed-up web services among Cloud Servers in order to make full use of their information collection and computing power in cross-sectional and/or longitudinal data analyses. Following is the pragmatic approach we took to building our pervasive on-line EEG-BCI system.

Contrast to popular belief, modern wireless sensors and mobile devices are no longer impoverished in their communication and computing capability. Both the Bluetooth® 4.0 protocol (Bluetooth Smart Technology: Powering the Internet of Things) and the IEEE 802.11n low-power Wi-Fi technology (Venkatesh) can support data transfer rates up to 24 Mb/s. Also, several low-power embedded processors have 32-bit processing units, floating point co-processors, direct memory access channels and power management units built into their system-on-chip (SoC) design. With these new technologies, the design decision now lies with the tradeoff between on-board computation and communication power budget. In fact, computation is usually more power efficient than communication unless the communication occurs over very short distance as in the case of Bluetooth personal-area networks. Cell phone communication is much less efficient as its power consumption increases in proportion to the *forth power* of the communication distance. With powerful embedded processors, the new generation of wireless sensors can perform various signal pre-processing tasks including artifact removal (Jung et al., 2000; Joyce et al., 2004), compressive sampling (Candes and Wakin, 2008), and even feature extraction (Suleiman and Fatehi, 2007) on board. These pre-processing tasks can transform large amount of raw data into compact representations and hence improve the combined power efficiency of computation and communication measured in Joule/bit. We have used these technologies to build a 10-DOF motion sensor (Zao et al., 2013), which consumes less electric power and supplies much more computing power than similar commercially available sensors.

Deploying ubiquitous Fog Servers close to the front-end devices (in terms of network distance) can serve two purposes at once: first, it can help the wireless sensors to provide sub-second real-time responses by offloading their heavy computation to the more powerful Fog Servers with minimal communication overhead, and it can also mitigate the communication bottleneck between the local area networks and the global Internet by drastically reducing the amount of traffic flowing between the Fog Servers and the Cloud Servers. In the example of our multi-player on-line EEG-BCI game, EEG Tractor Beam (section Multi-player On-line Interactive BCI Game), the Fog Servers sent only the brain states of individual players over the Internet every quarter of a second. Hence, the game generates very little real-time traffic even with hundreds of players participating in a single on-line session. Fragments of raw EEG data will be uploaded only after the game for the sake of building up the vast EEG data repository.

Computation off-loading becomes most effective when the Fog Servers possess high-performance multicore processors, are abundant in electric power and connected to both wired and wireless broadband networks. Game consoles are a perfect example of such servers. Other candidates include the television set-top boxes with Wi-Fi connectivity, the next-generation home Internet gateway with built-in servers and the dashboard computers on intelligent vehicles. Whenever the BCI frontends come within the wireless network coverage of these Fog Servers, they should connect themselves directly to these servers. They can then stream their data directly and perform real time signal processing and brain state prediction on these servers. The results can then be disseminated to the associated Cloud Server(s), the peer Fog Servers and the personal mobile devices in power and bandwidth efficient ways.

The Cloud Servers play both the roles of massive data repository and high-performance computing engine in our on-line EEG-BCI system. Nonetheless, not all these servers need to be installed in big data centers; many of them can be installed in server clusters all over the world. In fact, most data sets would likely be stored in local Fog Servers with only their meta-data uploaded onto the Cloud Servers. Together, the Cloud Servers create a logical Linked Data superstructure by maintaining a federated semantic meta-database and performing semantic search over this meta-database. Only when the semantic data search matches the meta-data with certain search criteria, the associated data sets will be transported to one or more Cloud Servers. Cross-sectional and/or longitudinal analyses will then be performed onto these data sets. Data will be cached within the Cloud Servers only for a finite duration; un-used data will be flushed so as to make efficient use of the cloud-based data storage.

#### Heterogeneous data interchanges

To ensure interoperability, our pervasive EEG-BCI system implements two Internet data interchanging mechanisms: (1) *machine-to-machine publish/subscribe data exchanges* between the sensors and the Fog Servers as well as among the peer Fog Servers; (2) *web-based client-server transactions* between the Fog Servers and the Cloud Servers.

The machine-to-machine publish/subscribe data exchanges are used to push multi-modal BCI data from the front-end sensors to one or more near-end Fog Servers. This data transport mechanism supports real-time multi-point communication with minimal overhead. We chose to use MQTT (Message Queuing Telemetry Transport) (IBM), a lightweight publish/subscribe protocol with reliable transmission, so that it can be implemented on simple low-power devices.

The client-server transactions enable the Fog Servers to interact with the Cloud Servers over a standard Web Service interface. We chose to employ RESTful Web Service (Fielding, 2000; Elmangoush et al., 2012), the de-facto standard server interfaces for mobile applications, to support these transactions. This choice ensures that our Fog Servers can interoperate with any web server in the Computing Cloud, and allows any user computer to query any of our Cloud Servers so as to obtain BCI services from our system.

#### Modularized software interfaces

Our pervasive EEG-BCI system aims at working with a garden variety of sensors as well as signal processing and neuro-imaging software. To do so, we must support conversion between different EEG data formats and provide program interfaces to software modules.

Currently, our system supports data conversion between the legacy BDF/GDF/EDF formats and the new Extensible Data Format (XDF) (Kothe, 2014b) as well as the SET format used by the MATLAB® EEGLAB toolbox (EEGLAB, 2014). Internally, our system employs Google protocol buffers (Protobuf) (Google, 2012) to en-code all the data sent through MQTT and RESTful protocols and uses Piqi (Lavrik, 2014) to convert the data between Protobuf, XML and JSON formats.

In order for our EEG-BCI system to work with several EEG analysis MATLAB® toolboxes including (BCI2000, 2014; BCILAB, 2014; EEGLAB, 2014), we developed an application program interface (API) between the MQTT publish/subscribe data transport protocol and the MATLAB toolboxes using the Lab Streaming Layer (LSL) middleware (Kothe, 2014a). This API supports data acquisition, time synchronization and real-time data access among MATLAB modules.

Finally, in order to enable the MATLAB toolboxes to interact with the Linked Data superstructure described in the next section, we also devised a RESTful Web Service interface to support semantic data up/downloading, redirection and search operations. This interface allows mobile applications (1) to add meta-data links to the streaming EEG data and/or the archived EEG data sets and (2) to perform semantic search over these data streams and data sets without knowing the details of the semantic data structure.

### Federated linked big data superstructure

The second technology supporting our pervasive on-line EEG-BCI system is a logical data superstructure that was constructed according to the W3C Linked Data guidelines (Berners-Lee, 2006). The sole purpose of employing the Linked Data technology is to enable the Fog and Cloud Servers as well as other authorized computers to perform *semantic data search* on a distributed repository of BCI data sets. Unlike human users, computers cannot tolerate ambiguity in the meanings of the keywords as they use these keywords to search for relevant sets or describe their characteristics. Traditional data models such as the relational model fail to deliver a proper solution as they lack the ability to specify the semantic relations existing among various data objects and concepts. We need a *semantic data model* and a *querying technique* that have rich semantics to describe the real-world settings of brain–computer interactions and provide sufficient granularity to specify different BCI stimuli and responses. In the following sections, we introduce briefly the principle behind the Linked Big Data Model we adopted and the Semantic Sensor Network (SSN) ontology we extended to support semantic search among the BCI data collection.

#### Semantic data model and linked big data

Linked Data (2014) is the latest phase of a relentless effort to develop a global interconnected information infrastructure: the first phase began with the deployment of the Internet, which connects information processors (computers) together using physical communication networks; the second phase was marked by the development of the World Wide Web, which connects information resources (documents and services) together through logical data references; the third and the latest phase was launched through the dissemination of Linked Data, which connects information entities (data objects, classes, and concepts) together via semantic relations. From another perspective, the migration from World Wide Web to Linked Data represents a paradigm shift from publishing data in human readable HTML documents to machine readable semantic data sets so that the machines can do a little more of thinking for us.

In essence, a Linked Data set is a graph with its nodes being the *data objects*, *classes*, and *concepts* while its edges specifying the *relations* among these data entities. Conforming to the convention of Semantic Web (W3C, 2014b), every relation in this graph is specified as a *predicate* in Resource Description Framework (RDF) (W3C, 2014a); each RDF predicate or triplet consists of a *subject*, an *object* and a *relation* all expressed in Extensible Markup Language (2013) format. The formal semantics of a Linked Data set is prescribed by a core sub-graph known as a *RDF schema*. It specifies the semantic relations between data classes, concepts and attributes that are relevant to the data set. The additional information superimposed onto the actual data is referred to as the *meta-data*. A RDF schema that encompasses all the data classes, concepts and relations in a field of knowledge is known as an *ontology*. This graphic depiction of semantic relations presents a *semantic data model* in *knowledge representation* (Randall Davis, 1993).

To find all the entities in a Linked Data set that are related in a specific data object, concept or an attribute, one simply perform a search or traversal through the graph: all the nodes that can be reached via the traversal by following a set of constraints constitute the results of this *semantic search*. Since the graph traversals can be performed by computers without any human, they suit perfectly for automatic machine-to-machine information query. A query language known as SPARQL (W3C, 2014c) was developed to specify the criteria (objectives and constraints) of semantic search based on RDF predicates much the same as SQL has done for the relational databases.

We adopted the approach of Linked Big Data (Dimitrov, 2012; Hitzler and Janowicz, 2013) to support machine-to-machine semantic search among BCI data sets. This approach requires us to deposit a layer of meta-data upon the BCI data sets. These meta-data annotate the data sets (as a whole and in parts) with *semantic tags* that describe the characteristics of the subjects, the circumstances and the mechanisms with which the BCI data have been captured. Semantic search based on these meta-data will enable computers to find the annotated data sets and/or their fragments that match specific search criteria. Unlike Big Linked Data, an alternative approach that converts every data entity into a Linked Data object, the Linked Big Data approach maintains the original data representation, but adds meta-data “tags” to the data sets in order to facilitate the semantic search.

Our colleagues at the Swartz Center for Computational Neuroscience (SCCN) have designed the meta-data tags for annotating EEG data sets. Among them, the *EEG Study Schema* (ESS, 2013) and the XDF (Kothe, 2014b) were devised to describe the *context* (subjects, circumstances and mechanisms) of the recording sessions. On the other hand, the *Hierarchical Event Descriptor Tags for Analysis of Event-Related EEG Studies* (HED) (Bigdely-Shamlo et al., 2013) was devised to specify the events that evoke the EEG responses. Our contribution includes the specification of a BCI Ontology, which captures the semantics of ESS and HED vocabulary, and the development of a RESTful Web Service interface for managing and querying the BCI repository.

#### BCI Ontology

A pre-requisite to organize BCI data sets according to the Linked Data guidelines is to devise a *BCI Ontology* to capture the BCI domain knowledge. Since brain–computer interactions can be regarded as a form of sensor activity, we decided to devise the BCI Ontology as an application specific extension to *SSN* Framework Ontology (W3C, 2011) for organizing the sensors and sensor networks on the World Wide Web.

**Figure 2:**
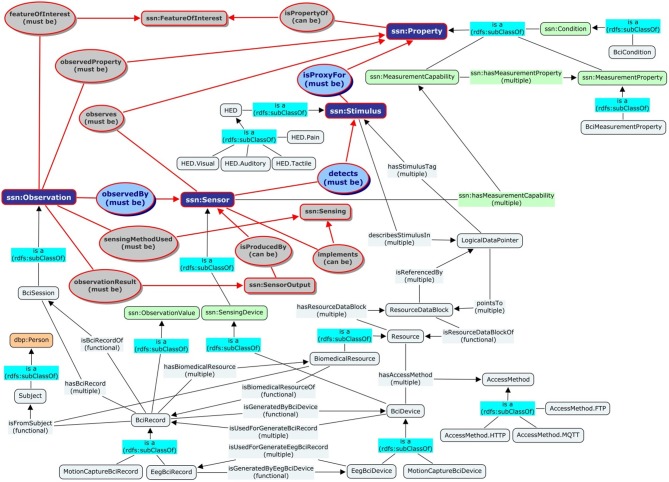
**Alignments between the proposed BCI Ontology and the SSN Stimuli-Sensor-Observation ontology design pattern**. The directed graph depicts the relations (edges) among the cores concepts/classes (rounded-square nodes) from different namespaces including the default BCI namespace (sky-blue colored nodes), the SSN namespace (colored nodes with ssn prefix), and the Dbpedia namespace (tan colored nodes with dbp prefix). The sub-graph with red outlines contains the basic SSN concepts. The rest of the graph shows how the concepts such as Subject, BciSession, BciRecord, BciDevice, Resource, and HED are aligned with the concepts of Stimuli, Sensor, and Observations (dark-blue nodes) in the design pattern. For example, the class BciDevice in the BCI namespace is a subclass of SensingDevice in the SSN namespace, which in turn is a subclass of Sensor in the SSN ontology design pattern.

The core of SSN Ontology is the *Stimulus-Sensor-Observation Ontology Design Pattern* (Compton and Janowicz, 2010) built upon the basic concepts of stimuli, sensor and observations. The sub-graph marked with the red outlines in Figure 2 is the semantic graph of this design pattern.

- **Stimuli:** these are the detectable changes in the environment that trigger the sensors to perform observations. BCI Ontology extends the concept of Stimuli by appending the *Hierarchical Event Descriptors (HED)* of all EEG stimulating events as its sub-classes.
- **Sensors:** these are the physical objects that perform observations. The design pattern makes a clear distinction between the object of sensors and the procedure of sensing. Sensors are the composite abstraction of sensing devices while the sensing procedures are the descriptions that specify how sensors should be realized and deployed in order to measure certain observable properties. In BCI Ontology, the concept of Sensor is extended by adding a BCI Device as a specialized concept of Sensing Device.
- **Observations:** these are multi-dimensional objects that capture information about the stimuli, the sensors, their outputs and the spatial-temporal specification of the sensing activity. In BCI Ontology, the concept of Observation is extended to include all Sessions of BCI activities. XDF and ESS supply the vocabulary. Among them, XDF specifies the recording types (such as EEG and Motion Capture) as well as the characteristics of human subjects, recording environments and experiment conditions. ESS, on the other hand, specifies sessions, recording modalities and event descriptions.

Following are some of the basic concepts/classes defined in the BCI Ontology namespace: http://bci.pet.cs.nctu.edu.tw/ontology#. They are aligned with the core concepts in the SSN Stimulus-Sensor-Observation Ontology Design Pattern. Figure 2 shows a few examples of the alignment.

- **Sessions, Resources, Devices, and Records:** these are the basic concepts and terminology pertained to BCI applications. Among them, Sessions align with Observations; Records align with Observation Values and have EEG Records as a subclass; Devices align with Sensing Devices, which has EEG Device being its subclass; Resources is an abstraction of data files and streams.
- **Stimulus HED Hierarchy Concepts:** as mentioned before, these conceptual descriptors represent the EEG stimulating events based on to the HED vocabulary. The first level notions of the stimuli events classification, includes: visual, auditory, tactile and pain descriptors.
- **Subjects:** these are people with certain attributes, on which the sessions are recorded. The concept is a synonym to *Patient* in the HL7 standard, which in turn was derived from the base class of *Person* in (DBpedia, 2014).
- **Access Methods and Protocols:** These concepts specify the protocol parameters for accessing the associated resources. Current access methods include MQTT for accessing real-time data streams, HTTP and FTP for data files.

#### Federated linked data repository and semantic search

In order to allow BCI users to maintain recorded data in their own servers as well as conducting semantic data search among multiple servers, our BCI system must be equipped with a distributed Linked Data repository and a federated semantic data querying scheme. Both of these facilities are safeguarded by Internet communication security and multi-domain attribute-based access control mechanisms.

The distributed Linked Data repository consists of two functional components: (1) the individual Fog/Cloud Servers that maintain the actual BCI data sets and (2) the RDF repository spread across the Cloud Servers that manage the meta-data of the Linked Big Data superstructure. In order to protect user privacy, all personal information and raw BCI data shall be stored in either the Fog Server(s) on users' premise or the trusted Cloud Server(s) authorized by the users. All sensitive data are protected by strong communication and information security measures. Only the anonymous subject identifiers, the universal resource identifiers (URI) and the meta-data tags of the data sets may be disseminated among the Cloud Servers. Together, the Cloud Servers maintain a distributed *RDF repository* that can be queried under anonymity protection using the *SPARQL Protocol and RDF Query Language* (SPARQL) v.1.1 (W3C, 2014c).

**Figure 3:**
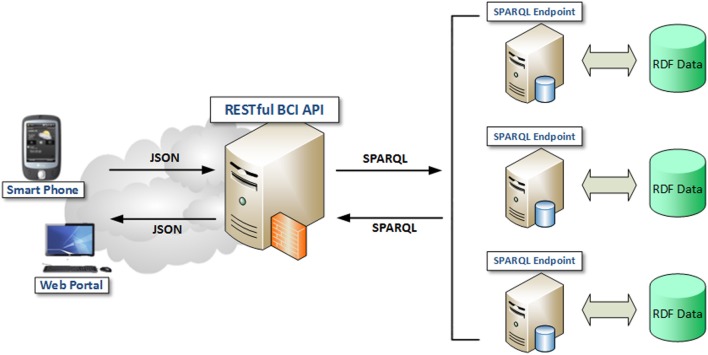
**Linked BCI Data Repository over a Federation of SPARQL Endpoints (Rakhmawati, 2013)**.

SPARQL 1.1 query language supports the *federation* of multiple SPARQL endpoints. As shown in Figure 3, a client can issue a SPARQL 1.1 query to a *query mediator*, which will convert the query into several *sub-queries* and forward them to different SPARQL endpoints. Each endpoint then processes the sub-query it received and sends back the query results. Finally, the mediator joins the query results from different endpoints to produce the final result.

Currently, we use *Virtuoso Universal Server* (VUS) v6.01 (OpenLink Software, 2014) to host the distributed RDF repository. Offered freely as a key component of (LOD2 Technology Stack, 2013), VUS is the most popular open-source semantic search engine for Linked Data applications. VUS can perform *distributed RDF link traversals* as a rudimentary mechanism to support federated SPARQL. To use this mechanism, we developed a Federated Query Mediator that can run on any Fog Server. This mediator can accept semantic data queries expressed in the RESTful/JSON web service format; transform them into SPARQL 1.1 sub-queries and then issue these sub-queries to the VUS installed in multiple Cloud Servers. This RESTful/JSON-compatible Federated Query Mediator not merely implements the federated semantic search; it also provides a standard web service interface for any authorized mobile applications to issue SPARQL queries and thus access our linked BCI repository.

## Results

### Pilot system

In the past two years, the Pervasive Embedded Technology (PET) Laboratory at NCTU and the SCCN at UCSD have been working together closely to develop a proof-of-concept prototype of the proposed pervasive EEG-based BCI system. In this endeavor, we chose to use wireless dry-electrode EEG headsets and MEMS motion sensors as the *front-end devices*, Android mobile phones as the personal user interfaces, compact personal computers as the *near-end Fog Servers* and a supercluster of computers hosted by the Taiwan NCHC as the *far-end Cloud Servers*. Table 1 provides a detail list of hardware and software components that are used to build this proof-of-concept pilot system.

**Table 1:** **Hardware and software components for the pervasive on-line EEG-BCI pilot system**.

**HARDWARE COMPONENTS**	
EEG headsets	MINDO-4S EEG Headsets
	Electrodes: 4 Soft Dry Forehead Mounted
	Sampling rate: 128 s/s
Motion sensors	BodyDyn-II 10-DOF Motion and Posture Sensors
	CPU: Atmel AT91SAM9G20 CPU
	Memory: 256 Mbytes NAND Flash and 64 Mbytes SDRAM
	Storage: 8 GB Micro-SD
	Radio: Atrie BTM-204B Bluetooth 2.1 EDR+
Mobile devices	Samsung Galaxy S3/Note 1 Smart Phones
	Samsung Galaxy Tablet
	Asus Transformer 1 Tablet
Fog Servers	Shuttle XPC-SH67H3 Compact Personal Computers
	CPU: Intel i7 Quad Core
	GPU: NVidia 550TI GPU
	Memory: 16 GB RAM
	Storage: 128 GB SSD Hard Disk
Cloud Servers	*Taiwan NCHC Supercluster*
	Cluster: Acer AR585 F1
	Processors: AMD Opteron 6174, 12 cores, 128 GB RAM
	FATs: AMD Opteron 6136, 8 cores, 2.4 GHz, 256 GB RAM
	OS: Novell SuSE Linux Enterprise 11 SP1
	LAN: 10 Gbps Ethernet
Cloud Servers	*UCSD SCCN VM Server*
	Processor: ProLiant DL380 G6
	Storage: MSA2312SA, 10TB RAID
	Virtual machine: VMware ESXi v.4.1.0
	OS: CentOS v.5.5
**SOFTWARE COMPONENTS**	
Fog Server OS	Ubuntu Linux v.13.10 Desktop
Computing platform	MATLAB R2013a
Parallel processing	NVidia CUDA v.5.0
Signal processing	BCILAB v.1.02b
Application interface	Lab Streaming Layer (LSL) v.1.05
Real-time messaging	Mosquitto MQTT v3.1 Publish/Subscribe Broker

**Figure 4:**
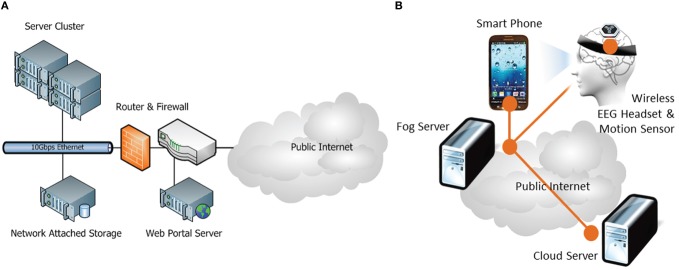
**Pilot system architecture of (A) Cloud Computing site at NCHC, Taiwan and (B) Fog Computing sites at NCTU PET Lab, Taiwan and UCSD SCCN, San Diego, California**.

This pilot system is currently deployed on two application/fog-computing sites: (1) NCTU PET Lab, (2) UCSD SCCN, and two cloud-computing sites: (1) NCHC supercluster and (2) UCSD SCCN virtual machine server. Figure 4 illustrates the system configuration at these sites. Both NCTU and UCSD fog-computing sites have participated in all pilot experiments and demonstrations. Currently, the NCHC cloud-computing site is hosting the BCI data repository and the BCI web portal while the SCCN server is maintaining an archive of legacy BCI data sets.

In the past year, both PET and SCCN teams have used this pilot system to perform different experiments demonstrating the capability and the potential of pervasive real-world BCI operations. Following subsections describe the two multi-site experiments we have performed.

### Synchronous BCI data streaming over internet

The NCTU-UCSD team performed a successful live demonstration of real-time synchronous multi-modal BCI data streaming at a project review meeting of the Cognition and Neuroergonomics Collaborative Technology Alliance (Can-CTA) Program on March 13, 2013. In that intercontinental demonstration, Prof. John Zao was wearing a four-channel wireless *MINDO-4S* EEG headset and a 9-DOF *BodyDyn* motion sensor at NCTU PET Lab in Hsinchu, Taiwan. Sampled data from both sensors were transmitted simultaneously via Bluetooth to a Samsung Galaxy Note 1 smart phone. The data streams were then sent to a Fog Server at the PET Lab and multicasted over the Internet to a Cloud Server at the NCHC also in Hsinchu, Taiwan and a desktop computer at UCSD SCCN in San Diego, California. Four-channel EEG data as well as 3D linear acceleration and 3D angular velocity—with a total of 10 channels—were displayed at SCCN in synchrony with the live image of Prof. Zao's movements that was beaming through a Google Hangout session. Almost no perceptible delay can be seen between the video images and the EEG/motion waveforms appeared on the display at SCCN. A video clip attached to this paper shows an excerpt of that demonstration session.

**Figure 5:**
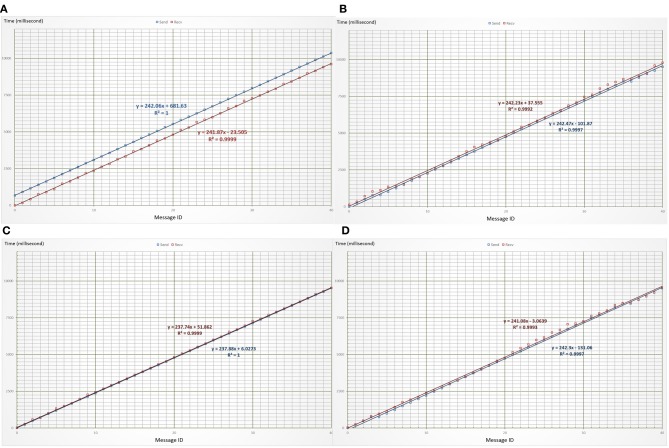
**Time traces of end-to-end synchronous transport of motion and EEG data streams. (A,B)** show the time traces of motion and EEG data transports in two separate sessions. **(C,D)** show the traces of both transports in the same session. The blue lines mark the traces of transmission time while the red lines mark those of reception time. Their slopes give the average transmission and reception intervals of individual messages.

Detail timing measurements of the end-to-end synchronous transports were made later in August during several replay of the demonstration and analyzed off time. Figure 5 shows the time traces of standalone and concurrent transport of the two data streams. Table 2 lists the formats and sizes of individual messages as well as the statistics of timing measurements of the transports. The significant differences in the mean values of transport latency were due to the offsets existing between the system clocks in the mobile phone at NCTU and the desktop computer at UCSD.

**Table 2:** **Performance measurements of synchronous BCI data streaming over Internet**.

**EEG DATA STREAM**	
Sampling rate	128 sample/second
Sample size	4 channels × 4 bytes (signed integer) = 16 bytes
Message size	32 samples + 2 bytes (MQTT Header) = 514 bytes (payload only)
Data rate	4 message/second = 2056 bytes/second (payload only)
Transport timing	*Standalone session*
	Interval: 242.2 ms (Tx)/242.5 ms (Rx)
	Latency mean^a^: 103.2 ms
	Latency Std. Dev.: 74.7 ms
	*Concurrent session*
	Interval: 241.1 ms (Tx)/242.3 ms (Rx)
	Mean^a^: 65.2 ms
	Standard Deviation: 59.9 ms
**MOTION DATA STREAM**	
Sampling rate	50 sample/second
Sample size	6 channels × 4 bytes (signed integer) + 8 byte (timestamp) = 32 bytes
Message size	13 samples + 2 bytes (MQTT Header) = 418 bytes (payload only)
Data rate	4 message/second = 1672 bytes/second (payload only)
Transport timing	*Standalone session*
	Interval: 242.1 ms (Tx)/241.9 ms (Rx)
	Mean^a^: −713.5 ms
	Standard Deviation: 42.2 ms
Transport timing	*Concurrent session*
	Interval: 237.4 ms (Tx)/237.9 ms (Rx)
	Mean^a^: 43.2 ms
	Standard Deviation: 32.0 ms

a *The average or mean values of transport latency were contaminated by the offset between the system clocks in the mobile phone at NCTU and the desktop computer at UCSD*.

These time traces show that no message was lost because the transport was conducted using MQTT messaging over TCP sessions. Small standard deviations of transport latency imply that few retransmissions were needed to provide reliable delivery. Latency of the EEG sessions fluctuates slightly more than that of the motion sessions; this suggests that a few more retransmissions were needed to deliver the longer EEG messages. The average transmission intervals (237–243 ms) in both standalone and concurrent transport sessions match closely with the expected quarter-second (250 ms) emission interval of the data messages. Besides, the average reception intervals also match closely with the average transmission intervals. These matching figures hinted smooth transmissions that were free of hop-by-hop traffic congestion and end-to-end message queuing. This superb performance may be partially due to the fact that the experiment was carried out between two university campuses equipped with gigabit Ethernets. Larger fluctuations in transmission/reception intervals as well as transport latency shall be expected when the data streaming is conducted over home networks.

Both the live demonstration and the performance statistics indicate that it is entirely possible to send BCI data streams reliably in real time to multiple destinations over the Internet. Thus, this experiment affirms the feasibility of Internet-based on-line EEG-BCI operation. Nonetheless, we must point out a potential *scalability* issue that may arise during multicasting of multi-channel EEG data streams. As the EEG channel numbers and sampling rates increase, the data rates of the multicasting sessions may quickly exceed the up-link bandwidth (approximately 1 Mbps) of home networks. In order to avoid causing network congestion in these cases, data compression techniques such as *compressive sampling* (Candes and Wakin, 2008) must be employed to reduce the message size. In fact, as a general principle, we should avoid sending raw data over the Internet in real time because such a practice will not only consume more network bandwidth but also incur longer transport latency. With the presence of ubiquitous Fog Servers, we should perform most real-time signal processing and brain state prediction on the Fog Servers and send only the extracted signal features, the brain states and the meta-data over the Internet in real time. This operation principle was demonstrated in the following experiment.

### Multi-player on-line interactive BCI game

In order to optimize the communication and computation efficiency, users of our pervasive EEG-BCI system should always use a Fog Server nearby to perform real-time signal processing and brain state prediction rather than performing the computation at the frontend sensors / mobile phones or sending the raw data over the Internet to the Cloud Servers. To demonstrate this operation principle, we developed the *EEG Tractor Beam*, a multiplayer on-line EEG-BCI game, and launched its first game session on September, 2013. Since then, this game has been played in several public occasions with players from both US and Taiwan.

**Figure 6:**
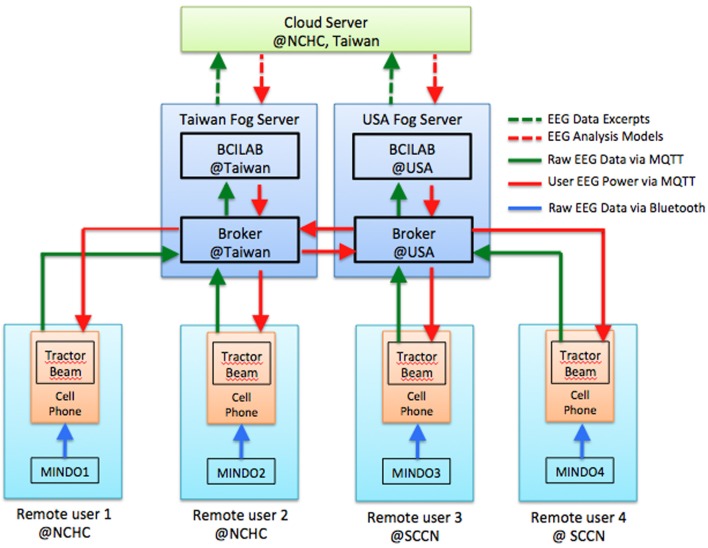
**Fog and Cloud Computing architecture for multiplayer on-line EEG-BCI game**.

Figure 6 illustrates the system architecture for this game, which is also a typical configuration for multi-site interactive BCI operation. Each user has a typical BCI frontend (shown as a sky blue box) consisting of an EEG headset and a mobile phone that are connected to a local Fog Server (a navy blue box). The Fog Servers associated with different users may exchange information with one another and a Cloud Server (the green box). The game was running as a mobile application on each user's mobile phone, which serves mainly as a graphic user interface (GUI). Raw EEG data streams were sent directly to the Fog Server or through the mobile phones. Real-time signal processing and prediction were performed on the Fog Servers, each of which ran a BCI signal processing pipeline. The brain states of individual users were published by the Fog Servers and sent to the game running on each mobile phone, which subscribed for the brain state information.

On its display, the multiplayer game shows all the players on a ring surrounding a target object. Each player can exert an attractive force onto the target in proportion to her level of concentration, which was estimated using the following formula (Eoh et al., 2005; Jap et al., 2009):

$$∁\;≜\;\ln\left(\frac{{\text{PSD}}_{\beta }}{{\text{PSD}}_{\alpha }+{\text{PSD}}_{\theta }}\right)$$

**Figure 7:**
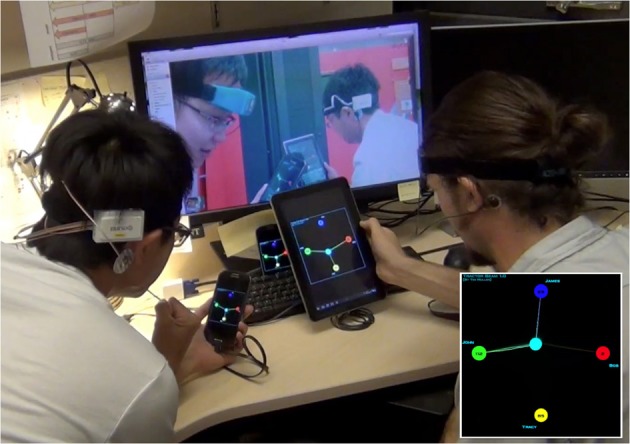
**An EEG Tractor Beam game session with four people playing over the Internet: two players at SCCN in San Diego, USA are shown in the foreground while two other players at NCTU in Hsinchu, Taiwan appear in the monitor display**. The inset at the lower right corner shows a captured view of the game display.

Where the PSDs are the average power spectral density in α, β and θ bands of the player. In order to win the game, a player should try to pull the target toward herself while depriving other players their chances to grab the target. The game implements a “winner-take-all” strategy: a player is awarded points at a rate proportional to the percentage of total attractive force she exerts on the target, which is calculated by dividing that player's concentration level by the sum of the levels among all the players. However, a player can only start to accumulate points if she contributes at least her fair share to the total sum. A tractor beam will appear between that player and the target when her concentration level passes that threshold. That was when she starts to cumulate her points. Figure 7 shows a picture of four players engaging in the game across the Pacific Ocean.

**Figure 8:**
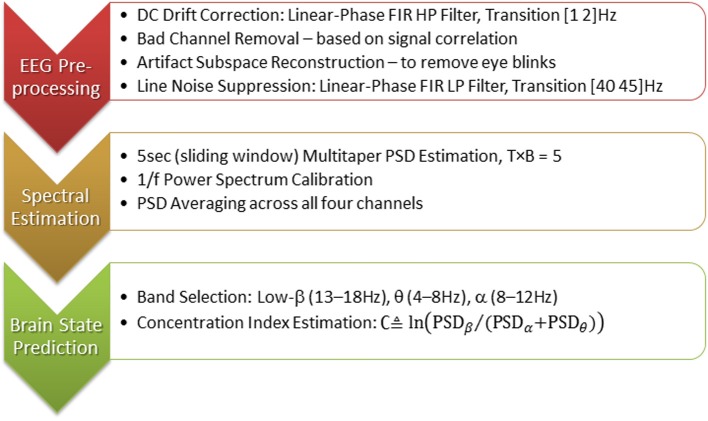
**Brain state estimation pipeline used in EEG Tractor Beam game**.

The necessary EEG signal processing and the estimation of *concentration level* ∁ were performed by the BCILAB/SIFT pipeline (Delorme et al., 2011) running on MATLAB R2013a (Mathworks, 2013) installed in the Fog Servers. Figure 8 displays the typical processing stages of this brain state estimation pipeline. Its MATLAB code was included in the Appendix for reference. The EEG preprocessing stage aims at cleaning up the raw EEG signals, which was heavily contaminated by artifacts due to eye blinks and head movements. The heavy computation of signal correlation and artifact subspace reconstruction (Mullen et al., 2012) can only be performed on the Fog Servers; these algorithms can quickly drain the batteries in the sensors and the mobile phones. Because players' concentration levels was estimated as the ratios between power spectral density in different EEG frequency bands, multitaper spectral estimation, power density calibration^1^ and averaging were done before the concentration levels were computed. Please note that although we chose to implement the BCI processing pipeline using BCILAB and SIFT, other real-time signal processing software can be used to perform the computation.

**Figure 9:**
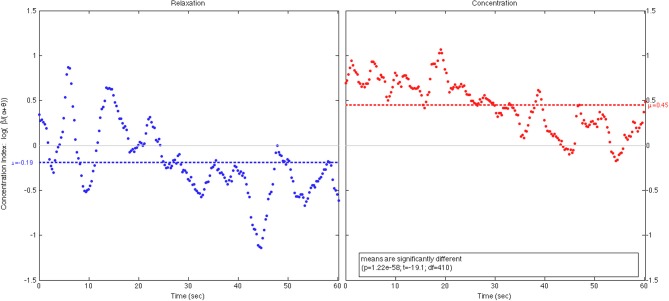
**The 1-min plots of a player's concentration level during a 2-min open- eye relaxation period (left) and an equal-length open-eye concentration period (right)**.

To demonstrate the working of our BCI processing pipeline, we showed in Figure 9 two 1-min scattered plots of a player's centration levels estimated during a 2-min open-eye relaxation period and an equal-length open-eye concentration period. The average concentration level during the relaxation period was μ_*R*_ = −0.19 < 0 as expected while the average level during the concentration period was μ_*C*_ = + 0.45. The difference between these values was statistically significant. The estimated values fluctuated notably during both periods. Partially, this was due to the wavering of player's concentration levels, but more likely, the fluctuations were caused by the remaining artifacts of head movements and muscle tension. These artifacts remain as an inevitable component of real-life EEG recording and a challenge to real-world BCI operation. Finally, both plots showed a general downward trend. This was because when the player tried to sustain her concentration, mental fatigue invariably set in after a short while; hence, her EEG power in beta band tended to decrease gradually relative to the power in alpha band. On the other hand, when the player tried to relax, it took some time for her to settle into a relaxed state; hence, we expect her alpha power to increase gradually relative to her beta power. In both cases, gradual decrease in concentration level was expected, especially if the player was untrained to perform the cognitive task.

In all the gaming sessions, the data rates and transport latencies over the Internet have been low since the Fog Servers published short messages merely containing players' identifiers and concentration levels. Also, the game displays among different players were synchronized because they all used Samsung Galaxy phones with compatible computing power. A small but noticeable display lag may appear if a player uses an old Android phone. This display lag can be eliminated using standard game synchronization protocols.

While *EEG Tractor Beam* is a somewhat frivolous demonstration of the capability of the pervasive on-line EEG-BCI system, it does demonstrate some powerful concepts that may have applications far beyond on-line gaming. Foremost, the system has the ability to acquire and process EEG data in real time from large number of users all over the world and feed their brain states back to these individuals as well as any professionals authorized to monitor their cognitive conditions. With distributed Fog and Cloud Servers, our on-line EEG-BCI infrastructure can be scaled indefinitely without adding unsustainable traffic load onto the Internet. Hence, it presents a viable way to realize *interact BCI*. Furthermore, the system has the ability to process, annotate and archive vast amount of real-world BCI data collected during the BCI sessions. Unlike the existing EEG databases, which depend on researchers to donate their data sets, this pervasive EEG-BCI infrastructure collects data sets—with users' approval—as an essential part of its normal operation. This intrinsic data collection provides a natural way to implement *“big data” BCI* as well as *adaptive BCI* in the near future. In the following section, we discuss the potential values and impacts of this pervasive on-line system toward the real-world BCI applications.

## Discussions

In this section, we examine the operation scenarios supported by the pervasive on-line EEG-BCI system as well as the costs and benefits of its potential use. This discussion begins with a comparison with the existing BCI systems and on-line physiological data repositories; it is concluded with a highlight of future development.

### Comparison with current practice

Currently, all BCI systems operate in a *standalone* fashion and need to be *personalized* before their use. No matter whether they are used to control patients' wheelchairs, conduct neuro-marketing or provide biofeedback, these systems require their users to go through tedious training processes in order to adapt them for personal use. Moreover, they often require the training process to be repeated once the use situations are changed. Our on-line EEG-BCI system, however, can download an initial brain state prediction model from the Cloud Server based on the real-world situation in which it operates, and then refine the model progressively using the data gathered from its users (section Adaptive BCI). This *adaptive* capability as well as its *interactive* and *big data processing* capability will distinguish our system from the existing ones.

The biomedical engineering community has been exploiting Cloud Computing and Big Data Mining technologies for years. In the past decade, several on-line physiological data repository including BrainMap (Research Imaging Institute, 2013), PhysioNet (Goldberger et al., 2000), and HeadIT (Swartz Center for Computational Neuroscience, 2013) have been put on line. Among them, PhysioNet earned the best reputation through the offering of a wide-range of data banking and analysis services. However, none of these data repositories are ready to accept real-time streaming data.

Furthermore, as demonstrated in the *EEG Tractor Beam* gaming sessions, our on-line EEG-BCI system also has the ability to support real-time multi-user collaborative/ competitive neuro-feedback. This unique ability may lead to many novel applications in cognitive collaboration, e-learning as well as on-line gaming and mind training.

### Operation scenarios

**Figure 10:**
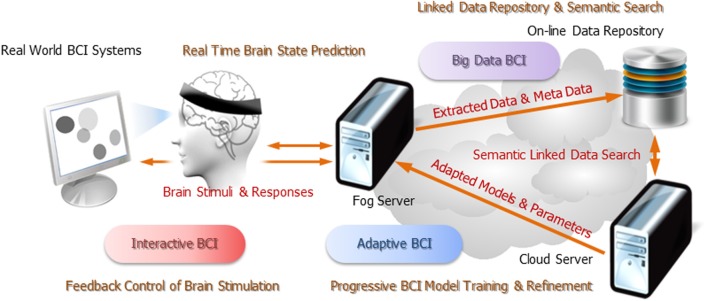
**Operation scenarios of pervasive EEG-BCI infrastructure**.

As shown in Figure 10, the pervasive on-line EEG-BCI system can operate in three different scenarios: Big Data BCI, Interactive (or Closed-Loop) BCI and Adaptive BCI. Each scenario represents an incremental enhancement of system capability.

#### Big data BCI

In this first operation scenario, the pervasive EEG-BCI system is endowed with the capability to collect multi-modal data along with relevant environmental information from real-world BCI applications anytime anywhere. This capability not only enables BCI applications to identify common EEG correlates among different users while they perform the same tasks or exposed to similar stimuli; it also provides a pragmatic way to gather vast amount of BCI data from real-life situations for cross-sectional and longitudinal studies. A linked BCI data repository and a RESTful web service API have been created for maintaining the data collection. Human clients would use the Web Portal (http://bci.pet.cs.nctu.edu.tw/databank) to access and query the data. Machine or application clients would use the RESTful web service API (http://bci.pet.cs.nctu.edu.tw/api) to perform specific data operations.

Currently, Big Data BCI is the only fully functioning scenario of our pilot system. All our experiments archived their data sets in the linked BCI data repository.

#### Interactive BCI

People's brain states and their EEG characteristics can be influenced acutely by the changes in environment conditions. Various visual, auditory, heat and haptic stimuli have long been used to evoke neural responses or modulate users' brain states. Currently, all these stimuli are static in nature as they lack the ability to adapt to users' changing brain states. Hence, the stimuli would become ineffective as habituation dampens users' neural responses or in the worse cases, cause harmful side effects.

Since the on-line EEG-BCI system can perform real-time brain state prediction on the Fog Servers, we can introduce a feedback control loops between the stimuli and the users' EEG responses. This *interactive* operation scenario can improve the accuracy of exogenous brain state prediction and the effectiveness of brain state modulation by applying the most powerful stimuli based on closed-loop feedback control.

#### Adaptive BCI

It is well known that people's EEG responses toward the same tasks (or stimuli) often differ significantly from one another and can change drastically over time. Thus, the prediction models employed by our BCI system must adapt to individual user's EEG responses and adjust their parameters continuously to track the changes of their characteristics. Usually, model adaptation and refinement are conducted using a large amount of training data. In order to reduce the amount of training data from individual users, we are exploring the feasibility of adapting the prediction model by leveraging the archived data collected from other users plus a small amount of training data acquired from this new user.

In our system, the adaptive BCI operation is performed through the cooperation between a Fog Server and its associated Cloud Server. The Fog Server will upload the annotated BCI data along with the predicted brain states, the prediction model specification and the confidence level on its prediction onto the Cloud Server. Then, the Cloud Server will issue semantic queries to find similar EEG data fragments among the archived BCI data sets and then apply *transfer learning* techniques on both the acquired and the archived data sets. Through repetitive trials, this *progressive refinement* process will likely produce a prediction model better-adapted to the BCI activity of that user in a specific real-world situation.

### Practical issues

Users are rightfully concerned about several practical issues such as *cost*, *availability*, *security* and *privacy* that may arise from the daily use of this elaborate infrastructure. Following are the concrete facts we hope may soothe some of these concerns.

First, the technologies we employ have already been used to provide Internet services today. The Cloud Servers have been running Google search and Yahoo web portals all along. Television set-top boxes and game consoles that function as the Fog Servers are popular electronic appliances. Almost without exception, mobile applications are installed in every smartphones these days. From this perspective, pervasive EEG-BCI is a natural outcome of the on-going trend to foster smart living using the state-of-art information and communication technologies. The incremental costs of using pervasive EEG-BCI will be quite affordable. A user only needs to purchase a wearable EEG headset and download a mobile application. The computing engine will be automatically downloaded onto her “fog server” once the user signs a service agreement. It is quite possible that pervasive EEG-BCI would become a fashion very much like the use of fitness gadgets these days.

Second, pervasive EEG-BCI will likely be offered by a supply chain of vendors that can bundle this service with Internet connectivity, content and computing. The huge infrastructure deployment and maintenance costs must be amortized among these service providers. Furthermore, the BCI data repository and the progressive model refinement technologies will take time to develop. Hence, this service must go through a maturing process.

Third, information security and personal privacy should indeed be users' common concerns. However, they must be dealt with as two separate issues. The basic guarantees of user anonymity, secure exchange, save storage and limited access can be provided through the employment of necessary communication and information security measures. These mechanisms are discussed in the following section. However, many users would be terrified by the notion that “the big brother can know not only where I click but also what I *think* when I browse the web!” Protection of personal privacy in that sense must be offered not merely through technical means but by developing and enforcing public policies according to social norms. Surprisingly, the protection of personal cognitive information is not more difficult than the protection of personal behavioral data collected by say Google, and is much easier than preventing information leakage via social networking because unlike individuals, reputable service providers are much more serious and diligent in guarding their clients' personal information.

### Future development

The pervasive EEG-BCI pilot system is merely a prototype. We plan to develop it into a field-deployable system within the coming year. Specifically, we will further develop its semantic data model and provide multiple ways to access streaming and archived data via multiple Internet protocols. Moreover, the following capability will be added to the system.

#### Cloud based progressive model refinement

Fog Servers will be able to perform adaptive brain state prediction with the aid of *progressive model refinement* carried out by the Cloud Servers. The process begins with *automatic annotation* of EEG data segments with their corresponding brain states according to the outcome of current prediction process. The meta-data annotation will be sent to the Cloud Servers so that cloud-based semantic search can find large number of data segments that match with certain personal, environmental and event specification. These data segments will then be fed into machine learning algorithms to calibrate the prediction model. The calibrated model will be pushed back to the Fog Servers and used to perform the next round of brain state prediction and data annotation. This iterative process will continue to improve the accuracy of prediction and enable the system to track the non-stationary brain dynamics. The Predictive Model Markup Language (PMML v.3.2, 2008; Guazzelli et al., 2009) will be adopted as the interoperable model specification and encoding format.

#### Information security and user privacy protection

We are developing a pervasive machine-to-machine communication security infrastructure based on the Internet standards: Host Identity Protocols (HIP) (IETF, 2014) and Host Identity Indirection Infrastructure (Hi^3^) (Nikander et al., 2004). HIP has become an increasingly popular approach to offer secure communication among the Internet of Things (Kuptsov et al., 2012). In addition, we developed a multi-domain attribute-enriched role-based access control architecture (Zao et al., 2014). Both of these technologies will be used to offer the essential communication and information security protection.

## Conclusion

The pervasive on-line EEG-BCI system we built culminated the development trends of two state-of-art information technologies: *Internet of Things* and *Cloud Computing*. As such, our pilot system can be regarded as a pioneering prototype of a new generation of real-world BCI system. As mentioned in section Operation Scenarios, these on-line systems will not merely connect the existing standalone EEG-BCI devices into a global distributed system; more importantly, they are fully equipped to support futuristic operations including intrinsic real-world data collection, massive semantic-based data mining, progressive EEG model refinement, stimuli-response adaptation. In academic and clinic research, these pervasive on-line systems will cumulate vast amount of EEG-BCI data and thus enable cross-sectional and longitudinal studies of unprecedented scale. Inter-subject EEG correlates of specific tasks and stimuli may be found through these studies. In the commercial world, numerous consumer applications will become feasible as wearable EEG-BCI devices can track people's brain states accurately and robustly in real time.

### Conflict of interest statement

The authors declare that the research was conducted in the absence of any commercial or financial relationships that could be construed as a potential conflict of interest.

## Footnotes

^1^The multitaper estimates of EEG power spectral density were multiplied by their sampled frequencies in order to compensate the natural decline of EEG spectral power inversely proportional to its frequency.

## References

[B1] BCI2000. (2014). Schalk Lab. Available online at: http://www.schalklab.org/research/bci2000

[B2] BCILAB. (2014). Swartz Center for Computational Neuroscience (SCCN). Available online at: http://sccn.ucsd.edu/wiki/BCILAB

[B3] Berners-Lee T. (2006). Linked DataŕDesign Issues. Available online at: http://www.w3.org/DesignIssues/LinkedData.html

[B4] Bigdely-Shamlo N. Kreutz-Delgado K. Miyakoshi M. Westerfield M. Bel-Bahar T. Kothe C. (2013). Hierarchical event descriptor (HED) tags for analysis of event-related EEG studies. Austin, TX: IEEE GlobalSIP Available online at: http://sccn.ucsd.edu/wiki/HED Retrieved November 2013, from Hierarchical Event Descriptor (HED) Tags for Analysis of Event-Related EEG Studies.

[B5] Bluetooth Smart Technology: Powering the Internet of Things. (n.d.). Available online at: http://www.bluetooth.com/Pages/Bluetooth-Smart.aspx

[B6] Bonomi F. Milito R. Zhu J. Addepalli S. (2012). Fog computing and its role in the internet of things, in Proceedings of the First Edition of the MCC Workshop on Mobile Cloud Computing (New York, NY: ACM), 13–16

[B7] Candes E. Wakin M. (2008). An introduction to compressive sampling. IEEE Signal Proc. Mag. 25, 21–30 10.1109/MSP.2007.914731

[B8] Compton M. Janowicz K. (2010). The Stimulus-Sensor-Observation Ontology Design Pattern and its Integration into the Semantic Sensor Network Ontology. Available online at: http://ceur-ws.org/Vol-668/paper12.pdf

[B9] Cummings M. L. (2010). VIEWS—technology impedances to augmented cognition. Ergon. Des. 18, 25–27 10.1518/106480410X12737888532804

[B10] DBpedia. (2014). wiki.dbpedia.org: About. Available online at: http://dbpedia.org/

[B11] Delorme A. Mullen T. Kothe C. Acar Z. A. Bigdely-Shamlo N. Vankov A. (2011). EEGLAB, SIFT, NFT, BCILAB, and ERICA: new tools for advanced EEG processing. Comput. Intell. Neurosci. 2011:130714 10.1155/2011/130714 21687590 PMC3114412

[B12] Dimitrov M. (2012). Semantic Technologies for Big Data. Amsterdam: XML Amsterdam

[B13] EEGLAB. (2014). Swartz Center for Computational Neuroscience (SCCN). Available online at: http://sccn.ucsd.edu/eeglab/

[B14] Elmangoush A. Magedanz T. Blotny A. Blum N. (2012). Design of RESTful APIs for M2M services, in 2012 16th International Conference on Intelligence in Next Generation Networks (ICIN) (Berlin: IEEE), 50–56 10.1109/ICIN.2012.6376033

[B15] Eoh H. J. Chung M. K. Kim S. H. (2005). Electroencephalographic study of drowsiness in simulated driving with sleep deprivation. Int. J. Ind. Ergon. 35, 307–320 10.1016/j.ergon.2004.09.006

[B16] ESS. (2013). Swartz Center for Computational Neuroscience. Available online at: http://sccn.ucsd.edu/wiki/ESS

[B17] Extensible Markup Language. (2013). W3C. Available online at: http://www.w3.org/XML/

[B18] Fielding R. T. (2000). Architectural Styles and the Design of Network-Based Software Architectures. Doctoral dissertation, University of California

[B19] Goldberger A. Amaral L. Glass L. Hausdorff J. Ivanov P. Mark R. (2000). PhysioBank, PhysioToolkit, and PhysioNet: components of a new research resource for complex physiologic signals. Circulation 101, 23 10.1161/01.CIR.101.23.e215 10851218

[B20] Google. (2012). Developer Guide—Protocol Buffers. Available online at: https://developers.google.com/protocol-buffers/docs/overview

[B21] Guazzelli A. Zeller M. Williams G. Lin W.-C. (2009). PMML: an open standard for sharing models. R. J. 1, 60–65 Available online at: http://journal.r-project.org/archive/2009-1/RJournal_2009-1_Guazzelli+et+al.pdf

[B22] Hitzler P. Janowicz K. (2013). Linked data, big data, and the 4th paradigm. Semantic Web J. 4, 233–235 10.3233/SW-130117

[B23] IBM (n.d.). Message Queuing Telemetry Transport. Available online at: http://mqtt.org/

[B24] IETF. (2014). Host Identity Protocol (hip). Available online at: http://datatracker.ietf.org/wg/hip/charter/

[B25] Jap B. T. Lal S. Fischer P. Bekiaris E. (2009). Using EEG spectral components to assess algorithms for detecting fatigue. Expert Syst. Appl. 36, 2352–2359 10.1016/j.eswa.2007.12.043

[B26] Joyce C. A. Gorodnitsky I. F. Kutas M. (2004). Automatic removal of eye movement and blink artifacts from EEG data using blind component separation. Psychophysiology 41, 313–325 10.1111/j.1469-8986.2003.00141.x 15032997

[B27] Jung T. P. Makeig S. Stensmo M. Sejnowski T. J. (1997). Estimating alertness from the EEG power spectrum. IEEE Trans. Biomed. Eng. 44, 60–69 10.1109/10.553713 9214784

[B28] Jung T. Makeig S. Humphries C. Lee T. M. Iragui V. Sejnowski T. (2000). Removing electroencephalographic artifacts by blind source separation. Psychophysiology 37, 163–178 10.1111/1469-8986.3720163 10731767

[B29] Kothe C. (2014a). Lab Streaming Layer (LSL). Available online at: https://code.google.com/p/labstreaminglayer/

[B30] Kothe C. (2014b). XDF (Extensible Data Format). Available online at: https://code.google.com/p/xdf/

[B31] Kuptsov D. Nechaev B. Gurtov A. (2012). Securing medical sensor network with HIP, in Wireless Mobile Communication and Healthcare, eds Nikita K. Lin J. C. Fotiadis D. I. Arredondo Waldmeyer M.-T. (Berlin; Heidelberg: Springer), 150–157 10.1007/978-3-642-29734-2_21

[B32] Lance B. J. Kerick S. E. Ries A. J. Oie K. S. McDowell K. (2012). Brain–Computer interface technologies in the coming decades. Proc. IEEE 100, 1585–1599 10.1109/JPROC.2012.218483018310804

[B33] Lavrik A. (2014). The Piqi Project. Available online at: http://piqi.org/

[B34] Linked Data. (2014). Connect Distributed Data Across the Web. Available online at: http://linkeddata.org/

[B35] LOD2 Technology Stack. (2013). Available online at: http://stack.lod2.eu/blog/

[B36] Mathworks. (2013). MATLAB: The Language of Technical Computing. (The MathWorks, Inc.) Available online at: http://www.mathworks.com/products/matlab/

[B37] Mullen T. Kothe C. Chi Y. M. Ojeda A. Kerth T. Makeig S. (2012). Modeling source dynamics and connectivity using wearable EEG, in IEEE EMB/CAS/SMC Workshop on Brain-Machine-Body Interfaces (San Diego).

[B38] Nikander P. Arkko J. Ohlman B. (2004). Host Identity Indirection Infrastructure (Hi3). Available online at: https://tools.ietf.org/html/draft-nikander-hiprg-hi3--00

[B39] OpenLink Software. (2014). Virtuoso Universal Server. Available online at: http://virtuoso.openlinksw.com/

[B40] PMML v.3.2. (2008). Data Mining Group. Available online at: http://www.dmg.org/pmml-v3-2.html

[B41] Rakhmawati N. U. (2013). Querying Over Federated SPARQL Endpoints—A State of the Art Survey. Galway: DERI, National University of Ireland

[B42] Randall Davis H. S. (1993). What is a knowledge representation? AI Mag. 14, 17–33

[B43] Research Imaging Institute. (2013). BrainMap. Available online at: http://www.brainmap.org/

[B44] Suleiman A. B. R. Fatehi T. A. H. (2007). Features Extraction Techniqes of EEG Signal for BCI Applications. Mosul: Faculty of Computer and Information Engineering, Department College of Electronics Engineering, University of Mosul

[B45] Swartz Center for Computational Neuroscience. (2013). HeadIT: Human Electrophysiology, Anatomic Data and Integrated Tools Resource. Available online at: http://headit-beta.ucsd.edu/

[B46] Venkatesh N. (n.d.). Ultra-Low Power 802.11n Wi-Fi – Wireless Connectivity for “The Internet of Things.” Available online at: http://www.low-powerdesign.com/article_redpine_100711.htm

[B47] W3C. (2011). SSN. Semantic Sensor Network Incubator Group. Available online at: http://www.w3.org/2005/Incubator/ssn/ssnx/ssn.html

[B48] W3C. (2014a). Resource Description Framework (RDF) and RDF Schema (RDFS). Available online at: http://www.w3.org/standards/techs/rdf#w3c_all/

[B49] W3C. (2014b). Semantic Web. Available online at: http://www.w3.org/standards/semanticweb/

[B50] W3C. (2014c). SPARQL Query Language for RDF (SPARQL). Available online at: http://www.w3.org/standards/techs/sparql#w3c_all

[B52] Zao J. K. Liao J. L. Hsu T. C. You C. Lu C. K. You C. K. (2013). BodyDyn—a 10-DOF body motion and posture monitoring system, Symposium on Engineering and Biology Applications (SEMBA 2013) (Tainan).

[B53] Zao J. K. Nguyen K. T. Wang Y. H. Lin A. C. H. Wang B. W. Liu J. W. S. (2014). Trustworthy emergency information brokerage service (TIBS). WIT Trans. Built Env. 133, 216–227 10.2495/DMAN130221

