# Supplementary material for: Pervasive brain monitoring and data sharing based on multi-tier distributed computing and linked data technology
Source: Front Hum Neurosci. 2014 Jun 3;8:370. doi: 10.3389/fnhum.2014.00370 (PMC4042686; doi:10.3389/fnhum.2014.00370)
Supplement: Supplementary file 1 [file DataSheet1.DOCX]

# Appendix: MATLAB Code for Estimating Subjects’ Concentration Level^[[1]](#footnote-1)^

% Define pipeline

opts = { ...

'FilterOrdering', { ...

'flt_clean_settings', 'flt_rescale' ...

'flt_fir' 'flt_fourier_bandpower'} ...

'DataCleaning', { ...

'DataSetting', {'1.1-beta' ...

'FlatlineRemoval', 'on' ...

'DriftCutoff', [1 2] ...

'BadChannelRemoval', 'on' ...

'ChannelDropoutRepair', 'off' ...

'BadSubspaceRemoval', { ...

'Cutoff', 10 ...

'ReferenceExtraction', { ...

'MaxBadChannels', 0.3 ...

'ParameterFitting', { ...

'StepSizes', [0.001 0.01]}}} ...

'BadWindowRemoval', 'off'}} ...

'Rescale', { ...

'ScaleFactor', 0.001} ...

'FIRFilter', { ...

'Frequencies', [40 45] ...

'Mode', 'lowpass' ...

'Type', 'linear-phase' ...

'StopbandRipple', -30 ...

'NormalizeAmplitude', true} ...

'FourierBandPower', { ...

'Filtering', { ...

'Representation', {'multitaper' ...

'TimeBandwidth', 5}} ...

'Bands', {[13 18], [4 12]} ...

'FreqCollapse', 'mean' ...

'AverageChannels', true}};

opts = {arg_guipanel('Function',@flt_pipeline,'Parameters',opts,'PanelOnly',false)};

% Load some calibration data (e.g. a short artifact-free segment)

calibData = exp_eval(io_loadset('data:\calibration.xdf'));

% Calibrate pipeline

cleaned_data = exp_eval(flt_pipeline('signal',calibData,opts{:}));

% Subscribe to MQTT topic and republish as local (named) LSL stream

... we do this by creating a timer object whose callback pulls ...

... new MQTT data and appends into LSL stream data buffer ...

... we create objects mindoPub and mindoSub for MQTT pub/sub ...

% Initialize online pipeline

pipeline = onl_newpipeline(cleaned_data,lsl_streamname);

% Main loop

while true

% pull data block from LSL stream and process through pipeline

[eeg_chunk,pipeline] = onl_filtered(pipeline,round(5*cleaned_data.srate));

% ... spectral power for [beta] and [alpha+theta] avg'd over channels

% ... now stored them in eeg_chunk.data(1) and eeg_chunk.data(2)

% compute log beta /(alpha+theta) power ratio

mqttData = log(eeg_chunk.data(1)./eeg_chunk.data(2));

% ... publish result over MQTT ...

mindoPub.updateMsg(num2str(mqttData(:)));

end

1. Published with MATLAB® 7.14 [↑](#footnote-ref-1)
